# Redox priming could be an appropriate technique to minimize drought-induced adversities in quinoa

**DOI:** 10.3389/fpls.2024.1253677

**Published:** 2024-04-04

**Authors:** Hassan Iqbal, Chen Yaning

**Affiliations:** State Key Laboratory of Desert and Oasis Ecology, Xinjiang Institute of Ecology and Geography, Chinese Academy of Sciences, Urumqi, China

**Keywords:** antioxidant activity, crop enhancement, drought, H2O2 application, osmoregulation, quinoa

## Abstract

The exogenous use of the redox compound (H_2_O_2_) plays a significant role in abiotic stress tolerance. The present study investigated various H_2_O_2_ application methods (seed priming, foliar spray, and surface irrigation) with varying concentration levels (0 mM, 5 mM, 10 mM, 15 mM, 40 mM, 80 mM, and 160 mM) to evaluate the efficiency of supplying exogenous H_2_O_2_ to quinoa under water-deficit conditions. Drought stress reduced quinoa growth and yield by perturbing morphological traits, leading to the overproduction of reactive oxygen species and increased electrolyte leakage. Although all studied modes of H_2_O_2_ application improved quinoa performance, surface irrigation was found to be sensitive, causing oxidative damage in the present study. Seed priming showed a prominent increase in plant height due to profound emergence indexes compared to other modes under drought conditions. Strikingly, seed priming followed by foliar spray improved drought tolerance in quinoa and showed higher grain yield compared to surface irrigations. This increase in the yield performance of quinoa was attributed to improvements in total chlorophyll (37%), leaf relative water content (RWC; 20%), superoxide dismutase (SOD; 35%), peroxidase (97%), polyphenol oxidase (60%), and phenylalanine ammonia-lyase (58%) activities, and the accumulation of glycine betaine (96%), total soluble protein (TSP; 17%), proline contents (35%), and the highest reduction in leaf malondialdehyde contents (MDA; 36%) under drought stress. PCA analysis indicated that physio-biochemical traits (proline, SOD, TSP, total chlorophyll, MSI, and RWC) were strongly positively correlated with grain yield, and their contribution was much higher in redox priming than other application methods. In conclusion, exogenous H_2_O_2_ application, preferably redox priming, could be chosen to decrease drought-induced performance and yield losses in quinoa.

## 1 Introduction

Future food production faces significant threats from climate change, urbanization, and the burgeoning growth of the population. Among these challenges, climate-change-induced water scarcity poses a major threat and exerts tremendous pressure on food production compared to other abiotic stresses. Enhancing crop performance under environmental stresses and adopting novel practices for sustainable agriculture can contribute to future food security (Muhie, 2022). Crops like quinoa, capable of enduring environmental challenges and offering higher nutritional values, have garnered significant global attention as a novel food crop. Quinoa, belonging to the *Chenopodiaceae* family, possesses a unique nutritional profile, including essential amino acids, vitamins, minerals, and micronutrients, surpassing other grain crops (Angeli et al., 2020; Khaitov et al., 2020). In addition, quinoa is an attractive option for agricultural diversification due to its exceptional ability to adapt to various abiotic stresses such as drought (Iqbal et al., 2018b; Khaitov et al., 2020; Mahdavi Rad et al., 2022; Abbas et al., 2023), salinity (Khaitov et al., 2020; Waqas et al., 2021; Afzal et al., 2022; Abbas et al., 2023), frost, and its ability to grow well even under marginal lands. Consequently, quinoa stands out as one of the rare crop plants that is naturally adapted to tolerate abiotic stress, thriving in dry conditions due to its low water requirements. Quinoa plants exhibit adaptive structural features, such as small, thin-walled cells, tissue flexibility, low osmotic potential, and dehiscence, enabling them to uphold leaf area and continue photosynthesis under water-deficit conditions (Jacobsen et al., 2009; Iqbal et al., 2018a; Abbas et al., 2023). Osmotic adjustment, a crucial mechanism contributing to drought tolerance in quinoa, involves efficient use of inorganic ion build-up (e.g., Ca^+^, K^+^, and Na^+^) and improved organic element synthesis (carotenoids and proline) under water-deficit conditions, setting it apart from other crops (Jacobsen et al., 2009; Iqbal et al., 2018b; Mahdavi Rad et al., 2022).

Despite these remarkable features, various studies have shown that drought stress markedly reduces the performance of quinoa, inhibiting its growth traits under severe drought stress (Iqbal et al., 2018a, Iqbal et al., 2018b; Huan et al., 2022; Mahdavi Rad et al., 2022; Abbas et al., 2023). Drought stress causes a significant reduction in plant growth and yield; however, its deleterious impact on crop performance could be ameliorated through the exogenous use of plant growth regulators, minerals, leaf extracts, and stress signaling molecules such as H_2_O_2_, NO, and H_2_S, thereby increasing stress tolerance (Terzi et al., 2014; Hossain et al., 2015; Savvides et al., 2016; Farooq et al., 2017; Iqbal et al., 2018b; Khan et al., 2019; Zhou et al., 2020; Habib et al., 2021; Chattha et al., 2022; Song et al., 2023). Hydrogen peroxide (H_2_O_2_), the most stable and long-lived redox molecule, rapidly diffuses across subcellular membranes and plays a dual role in plant metabolism. At higher concentrations, H_2_O_2_ is detrimental to biological membranes, causing programmed cell death; however, at normal concentrations, it regulates plant metabolism and facilitates other molecules in cellular signaling. Recent research indicates that H_2_O_2_ has the potential to improve antioxidant potential in crop plants under various environmental challenges, both biotic and abiotic stress conditions (Hossain et al., 2015).

Fewer studies have furthermore described that the exogenous use of H_2_O_2_ increases drought resilience in soybean (Guler and Pehlivan, 2016), wheat (Farooq et al., 2017; Habib et al., 2021), maize (Terzi et al., 2014; Ashraf et al., 2015), and quinoa (Iqbal et al., 2018a, Iqbal et al., 2018b). These studies primarily assess performance on the physiological basis of short-term growth experiments. However, from a sustainable agriculture perspective, there is a need to enhance both grain yield and quality, particularly for quinoa under drought conditions. It is important to note that H_2_O_2_ can be detrimental at higher concentrations, and researchers have predominantly focused on its impact in short-duration experiments due to its rapid diffusion.

Therefore, this study aimed to examine the influence of H_2_O_2_ applied through seed priming, foliar spray, and surface irrigation to identify the most suitable strategy for improving quinoa growth and yield performance under drought stress. This study represents, perhaps, the first detailed investigation in which various H_2_O_2_ application methods have been evaluated to enhance crop performance under drought stress. The hypothesis was that the exogenous application of H_2_O_2_ might improve the accumulation of compatible solutes, enhance secondary metabolites, and activate antioxidant enzymes, thereby increasing quinoa growth and yield under drought stress.

## 2 Materials and methods

### 2.1 Experimental setup

The present study was conducted in a rain-controlled wire house under natural conditions. Throughout the experimental period, the average day and night temperatures were recorded at 27.0°C and 19.2°C, respectively. The relative humidity was 59%, sunshine duration averaged 7.1 h, and precipitation measured 1.6 mm. Quinoa genotype ‘Pichaman’ was chosen as the experimental material, and 10-L plastic pots filled with a mixture of peat and vermiculite (in a 2:1 ratio) were utilized. The quinoa nursery comprised 200 plastic pots, each planted with five sterilized ‘Pichaman’ seeds. After 15 days of sowing, the plant population was maintained at one plant per pot and irrigated equally at 75% water holding capacity (WHC) until the 45th day of nursery sowing. Fertigation was carried out using the Hoagland solution, applied to all pots at 10–15 days’ intervals until maturity.

#### 2.1.1 Preliminary H_2_O_2_ concentrations test

**Figure 1:**
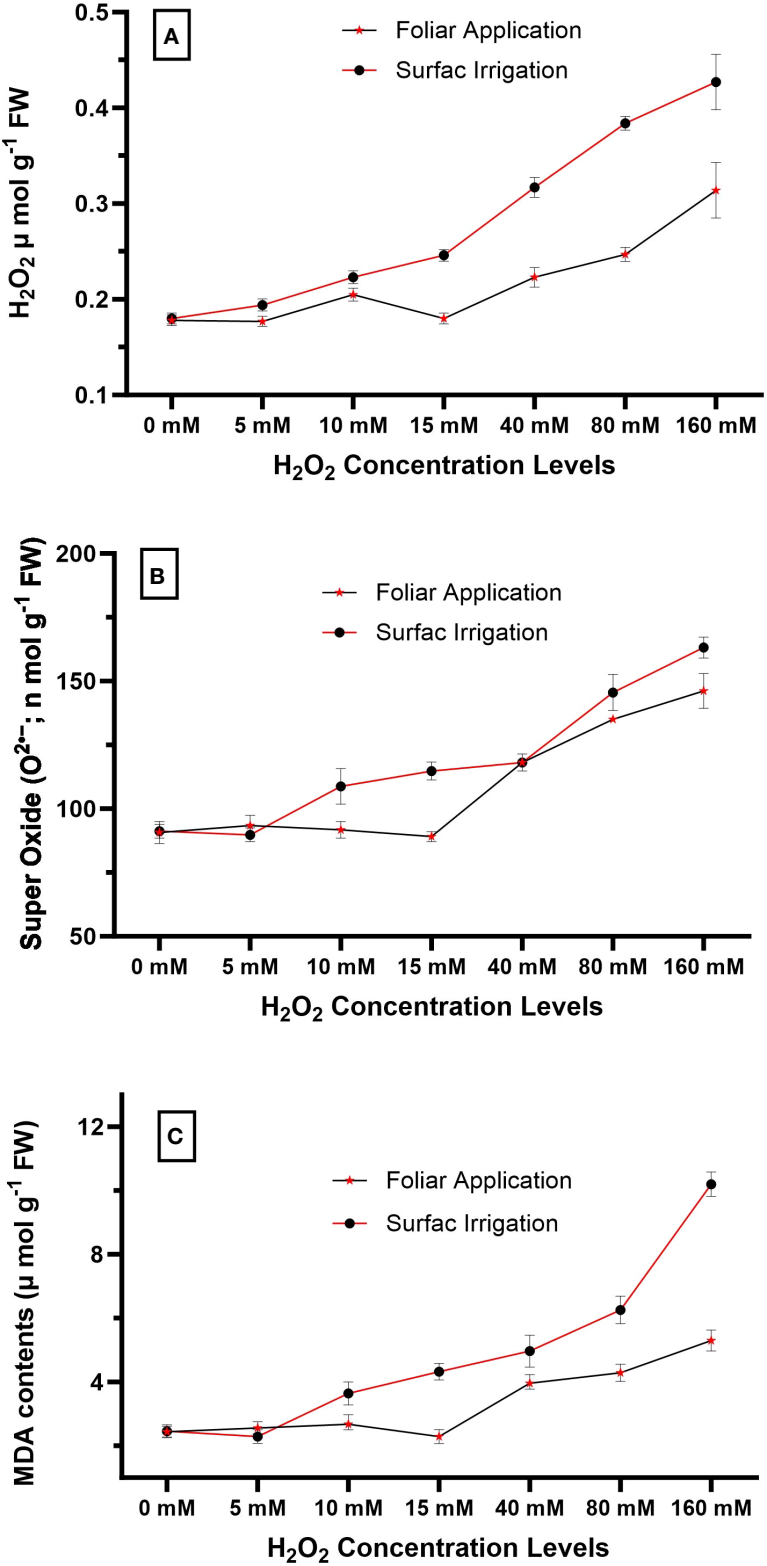
Impact of different levels of hydrogen peroxide concentration on **(A)** endogenous H_2_O_2_ concentration (µmol g^−1^ fresh weight), **(B)** super oxide O_2_^•−^ (n mol g^−1^ FW), and **(C)** MDA contents (µmol g^−1^ fresh weight) of quinoa seedlings. Values are the means ± SE of four replicates.

Before the major experiment, a preliminary test was conducted to determine the most suitable concentration of H_2_O_2_ for detailed investigation. In this test, 42 healthy quinoa seedlings (21 days old) were taken from the nursery and divided into two groups. Different levels of H_2_O_2_ concentrations (0 mM, 5 mM, 10 mM, 15 mM, 40 mM, 80 mM, and 160 mM) were used to treat the seedlings in group 1 (as foliar spray) and group 2 (as surface irrigation). Treatments were applied on the 21st and 28th days after sowing, and 1 week later (35th day), leaf samples were taken for the measurement of oxidative damages. Results revealed that foliar spray at 15 mM and surface irrigation at 5 mM H_2_O_2_ concentration showed the minimum oxidative damages in terms of MDA contents (**Figure 1**). Therefore, these concentration levels, 5 mM and 15 mM, were used for surface irrigation and foliar spray, respectively, in the detailed experiment.

#### 2.1.2 Redox priming and treatment selection

For redox priming, an 80 mM H_2_O_2_ solution, based on previous studies (Iqbal et al., 2018a), was selected and used as seed priming to evaluate emergence attributes compared to control quinoa seeds. During nursery transplantation for this investigation, 20 pots were separated and divided into two groups. One group consisted of control seeds (CK), and the second group consisted of primed seeds (H_2_O_2_). Five quinoa seeds were sown in each pot, and emergence counts were recorded daily until constant germination. Emergence attributes were calculated from the obtained data, and the results are presented in **Table 1**. After 45 days of nursery sowing, pots containing healthy seedlings from the control and 80 mM seed-primed set were selected for the detailed experiment. The study aimed to determine the impact of the redox compound (H_2_O_2_) via different application methods: seed priming, foliar spray, and surface irrigation on the performance of quinoa grown under drought stress conditions. The following six treatments were replicated four times under a randomized complete block design (RCBD), with four pots in each treatment (**Table 2**).

**Table 1:** Time taken to 50% emergence (E_50_; days), mean emergence time (MET; days), coefficient of emergence velocity (CVE; % day^−1^), emergence index (EI; % day^−1^) and final emergence percentage (FEP; %) influenced by seed priming (H_2_O_2_) as compared to control in quinoa.

Treatments	E_50_	MET	CVE	EI	ERT	FEP
Control (CK)	7.55 ± 0.29	7.39 ± 0.19	13.55 ± 0.30	1.23 ± 0.07	9.47 ± 0.31	68% ± 1.16
Seed priming (H_2_O_2_)	4.71 ± 0.42	4.79 ± 0.16	20.92 ± 0.68	2.63 ± 0.15	19.89 ± 0.93	85% ± 2.91

Values are the means ± SE of four replicates.

**Table 2:** Treatment description.

**Abbreviation**	**Treatment Description**
CK	Plants grown under sufficient water supply with no H_2_O_2_ treatment
DS	Plants grown under drought stress with no H_2_O_2_ treatment
DS+WS	Plants grown under drought stress were treated with the foliar spray of distilled water. (This treatment included as parallel control for the foliar treatment with H_2_O_2_)
DS+SP	Plants grown under drought stress were emerged from H_2_O_2_-primed seeds
DS+FS	Plants grown under drought stress were treated with the H_2_O_2_ solution (15 mM) via foliar spray
DS+SI	Plants grown under drought stress were treated with the H_2_O_2_ solution (5 mM) via surface irrigation

### 2.2 Imposition of drought stress and H_2_O_2_ treatments

Control pots were irrigated either daily or on alternate days to maintain 75% of water holding capacity (WHC). Drought stress was imposed 45 days after nursery sowing (DAS) when the seedlings were fully established. Drought was induced through deficit irrigation, using 50% of the water from control irrigation. To ensure the appropriate WHC, pots were regularly weighed (daily or on alternate days), and the required volume of water was used to irrigate each pot until maturity. To achieve greater accuracy, additional plants were grown to measure the weight of the growing plants at different developmental stages. For redox priming, seeds were soaked in an aerated solution of H_2_O_2_ (80 mM) at room temperature (25°C ±1) for 2 h and then re-dried to their original weight under shade. Foliar and surface irrigation treatments were applied at 45 days, 60 days, and 75 days after sowing (DAS). Distilled water (DW) was used as a control, applied in an equal amount as used for H_2_O_2_ application. For foliar application, a compression hand sprayer with a 2-L capacity was used to ensure an even distribution of the H_2_O_2_ solution on quinoa foliage. In surface irrigation, the required volume of water for deficit irrigation was replaced with an equal volume of 5 mM H_2_O_2_ solution (prepared with DW). Leaf samples for biochemical analysis were collected after 90 DAS. At maturity, plant height was measured, and harvesting was carried out.

### 2.3 Emergence indexes

The emerged seeds were counted on a daily basis until a constant emergence was observed. From the emergence tallies, various emergence attributes, including the coefficient of emergence velocity (CVE), emergence rate index (ERI), emergence index (EI), time taken to 50% emergence, mean emergence time (MET), and final emergence percentage (FEP), were calculated as per the method described by Kader (2005).


$$CVE=\sum N_{i}/\sum \left(N_{i}T_{i}\right)\times 100$$

$$MET\left(d\right)=\sum \left(N_{i}T_{i}\right)/\sum N_{i}$$

EI=(10∗n1)+(9∗n2)……..(1∗n10)

$$E_{50}\left(d\right)=ti+\left(N/2-ni/nj-ni\right)\;\left(tj-ti\right)$$

$$ERI(\%\;d^{-1})=\sum N_{i}/I$$

FEP(%)=(Total number of emerged seedlings/total number of seeds sown)×100

where *N_i_* (seeds emerged on day i), *T_i_* (days from sowing), *N* (is final emergence count), and *n_i_* and *n_j_* are cumulative number of emerged seeds at adjacent days *t_i_* and *t_j_* when *n_i_* < (*N* + 1)/2< *n_j_*.

### 2.4 Chlorophyll and carotenoids determination

For the estimation of chlorophyll, *a*, *b*, and carotenoid contents (CTDs), a quinoa leaf sample (0.25 g) was ground in an 80% acetone solution and calculated according to Arnon’s method (1949). The absorbance of the filtrate for chlorophyll *a*, chlorophyll *b*, and CTD was measured at wavelengths of 663 nm, 645 nm, and 480 nm, respectively, using a spectrophotometer (Cary 60; Agilent, USA).

### 2.5 Leaf relative water contents

The uppermost fully expanded leaves were destructively sampled for relative water contents (RWC) measurements. Leaf disks weighing 0.5 g fresh weight (W_F_) were immersed in double-distilled (DD) water. After 24 h, saturated disks were taken, and their turgid weight (W_T_) was calculated. Then, the dry weight (W_D_) was determined by drying saturated leaves at 65°C for 72 h, and RWC was calculated as follows:


$$\text{RWC }\left(\%\right)=\;\frac{W\text{F}-W\text{D}}{W\text{T}-W\text{D}}\;\times 100$$

### 2.6 Measurement of redox compounds (H_2_O_2_, O_2_^•−^)

#### 2.6.1 Hydrogen peroxide (H_2_O_2_)

The protocol defined by Velikova et al. (2000) was used to measure H_2_O_2_ concentration. The leaf sample (0.25 g) was ground in 3 mL of 5% TCA with 0.1 g charcoal and then centrifuged at 12,000×*g* for 15 min. Supernatant absorbance was observed at 390 nm, and endogenous H_2_O_2_ level was expressed as μmol g^−1^ FW.

#### 2.6.2 Superoxide radical (O_2_^•−^)

Superoxide radical (O_2_^•−^) was estimated according to Elstner and Heupel (1976) with slight modifications. A leaf sample of 0.3 g and 3 mL of a potassium phosphate buffer solution (65 mM; pH 7.8) were homogenized and centrifuged at 5,000×*g* for 10 min at 4°C. The supernatant, along with 65 mM potassium phosphate buffer, was mixed with hydroxylamine hydrochloride (10 mM) and then incubated at 25°C for 20 min. After incubation, the mixture was combined with sulfanilamide (17 mM) and α-naphthylamine (7 mM) and incubated again for 20 min at 25°C. Subsequently, ethyl ether was added and thoroughly vortexed. Optical density (OD) was later measured spectrophotometrically at 530 nm, and the O_2_^•−^ formation rate was calculated using NaNO_2_ standard curves.

### 2.7 MDA contents

Malondialdehyde (MDA) contents, byproducts of lipid peroxidation, were assessed in quinoa leaves using the TBA reaction method as defined by Dhindsa et al. (1981).

### 2.8 Membrane stability index

For the determination of the membrane stability index (MSI), leaf samples were collected in a test tube containing 10 ml of double-distilled water and placed in a water bath. After 30 min, the conductivity (EC1) of the test tube at 40°C was calculated using an EC meter. The second tube was heated in a water bath at 100°C. After 10 min, the EC2 was recorded, and the MSI was calculated using the following equation (Sairam and Saxena, 2000).


$$MSI=1-\frac{EC1}{EC2}\;\times 100$$

### 2.9 Glycine betaine contents

Quinoa leaf samples were extracted in 5 mL of warm double-distilled water (70°C). Then, 1 mL of the extract was added to 2N H_2_SO_4_ (1 mL) and potassium triiodide (200 μL) in a test tube. These substances were thoroughly shaken and chilled at 4°C for 60 min in an ice bath. Afterward, chilled double-distilled water (2.8 mL) and 1,2-dichloroethane (6 mL) were added, forming two layers in the mixture. The upper layer was discarded, and the optical density of the organic layer was read at 365 nm (Grieve and Grattan, 1983).

### 2.10 Total soluble protein and proline contents

Total soluble protein and proline contents of quinoa leaves were estimated via methods described by Bradford (1976) and Bates et al. (1973), respectively.

### 2.11 Antioxidant enzymes assays

#### 2.11.1 Leaf extract preparation

For extract preparation, crushed leaf sample (500 mg) was taken and ground with 2 mL extracting buffer then mixture was centrifuge at 15,000×*g* for 15 min. After centrifugation, the supernatant was collected and stored at −20°C for further measurements.

#### 2.11.2 Antioxidant enzymes

Superoxide dismutase (SOD) enzyme activity was assessed by observing the photo-reduction of nitroblue tetrazolium, while peroxidase (POD) activity was determined based on guaiacol oxidation as an electron donor, as defined by Chance and Maehly (1955). The activity of phenylalanine ammonia lyase (PAL) and polyphenol oxidase (PPO) enzymes was estimated following the protocols described by Siriphanich and Kader (1985) and Gauillard et al. (1993), respectively.

### 2.12 Agronomic and yield-related traits

At maturity, various growth parameters, including plant height, root length, and fresh and dry root weight per plant, were measured. Panicles were manually threshed, and seeds were collected to measure grain yield per plant. A subsample of healthy seeds was taken randomly; 100 seeds were counted and weighed on an electrical balance. After harvesting, shoot dry weight was recorded and added to grain weight for total biomass estimation. Later on, the harvest index (HI) was calculated as follows.


HI=Grain yield/biological yield

### 2.13 Statistical analysis

The study was laid out in a randomized complete block design (RCBD) with four replicates for each treatment. Replicated data collected from each treatment were statistically analyzed using the analysis of variance technique (ANOVA) and presented in tables and figures as mean (n=4) ± standard error (SE). Later on, the LSD test (least significant differences) at a 5% probability level was used to check significance between treatment means. For statistical analysis, STATISTIX-8.1 was employed, and for correlation analysis with the final graphical presentation (figures, tables, etc.), OriginPro 2023b software was used.

## 3 Results

### 3.1 Effect of H_2_O_2_ seed priming on emergence attributes

The results showed that seed priming with H_2_O_2_ applied at an 80 mM concentration significantly improves the emergence attributes in quinoa compared to control seeds (**Table 1**). MET and the time taken to 50% emergence (E50) were considerably reduced from 7.39 ± 0.19 to 4.79 ± 0.16 and 7.55 ± 0.29 to 4.71 ± 0.42, respectively, compared to control seeds. Moreover, the maximum final emergence percentage with higher CVE, ERI, and emergency index was found in H_2_O_2_-primed seeds compared to the control in the present study (**Table 1**).

### 3.2 Effect of different exogenous levels of H_2_O_2_ on oxidative damages

The generation of ROS (H_2_O_2_, O_2_^•−^) and MDA contents were measured at different levels of exogenous H_2_O_2_ concentration, and the results revealed that the 0–15 mM concentration range was found to be safe. However, surface irrigation and foliar application showed minimum MDA contents at 5 mM and 15 mM concentration, respectively (**Figure 1**). Endogenous H_2_O_2_ and superoxide O_2_^•−^ levels gradually increased with higher exogenous H_2_O_2_ concentrations, ultimately resulting in maximum oxidative damages at the 160 mM concentration, both in foliar and surface irrigation methods. Among the application methods, surface irrigation with H_2_O_2_ was found to be more sensitive compared to foliar application in terms of oxidative damages in the present study (**Figure 1**). As a result, the authors selected the 5 mM and 15 mM concentrations for surface irrigation and foliar application in the detailed experiment, respectively.

### 3.3 Effects on plant growth and photosynthetic pigments

Plant growth and photosynthetic pigments of quinoa markedly decreased (p ≤ 0.05) under drought stress (**Tables 3**, **4**). Plant height, dry weight, root fresh weight, root dry weight, chlorophyll a, chlorophyll b, total chlorophyll (a+b), and carotenoids decreased by 27%, 33%, 9%, 27%, 39%, 49%, 42%, and 36%, respectively, under drought stress relative to the control. Hydrogen peroxide (H_2_O_2_) applied as seed priming and foliar application were found to be efficient techniques to improve these growth attributes under drought stress compared to non-treated and water-treated plants. Drought-induced reduction in chlorophyll *b*, a + b, plant height, root fresh weight, root dry weight, and plant dry weight was recovered maximally in seed-primed plants, while foliar application of H_2_O_2_ showed the maximum chlorophyll *a* and carotenoid contents with non-significant improvement compared to H_2_O_2_ seed priming. However, in contrast to the control group, there was a significant improvement in root length observed in drought-stressed plants, further enhanced by H_2_O_2_ seed priming. Furthermore, surface irrigation showed statistically non-significant improvement in growth attributes compared to non-treated and water-sprayed plants under drought stress.

**Table 3:** Impact of different hydrogen peroxide applications methods on photosynthetic pigment concentrations (mg g^−1^ FW) in quinoa leaves under drought stress.

Treatments	Chl. *a*	Chl. *b*	Chl. (*a + b*)	Carotenoids
CK (Control)	0.94 a	0.39 a	1.33 a	0.42 a
DS (Drought stress)	0.57 c	0.20 d	0.77 d	0.27 c
DS + Water spray (DD)	0.59 c	0.21 d	0.80 d	0.27 c
DS + Seed priming (H_2_O_2_)	0.72 b	0.29 b	1.02 b	0.32 b
DS + Foliar spray (H_2_O_2_)	0.74 b	0.26 c	1.00 b	0.33 b
DS + Surface irrigation (H_2_O_2_)	0.63 c	0.24 c	0.87 c	0.28 c
**LSD (p ≤ 0.05)**	0.07	0.02	0.07	0.035

Values are the means of four replications and different letters indicating significant difference at p ≤ 0.05.

**Table 4:** Impact of different hydrogen peroxide applications methods on plant height (PH), root length (RL), root fresh weight (RFW), root dry weight (RDW), plant dry weight (PDW), grain yield (GY), 100 grain weight (100-GW), and harvest index (HI) of quinoa under drought stress.

Treatments	PH (cm)	RL (cm)	RFW (g)	RDW (g)	PDW (g)	GY (g)	100-GW (mg)	HI
CK (Control)	98.75 a	34.23 d	18.45 b	2.08 b	24.11 a	15.48 a	272 a	0.39
DS (Drought stress)	72.50 d	35.17 bc	16.85 e	1.51 e	16.04 d	10.39 c	203 d	0.39
DS + Water spray (DD)	72.25 d	35.19 bc	16.82 e	1.58 de	16.12 d	10.55 c	205 d	0.39
DS + Seed priming (H_2_O_2_)	81.75 b	37.05 a	18.90 a	2.25 a	18.82 b	13.38 b	217 bc	0.41
DS + Foliar spray (H_2_O_2_)	78.00 c	35.59 b	18.00 c	1.90 c	18.47 bc	12.92 b	220 b	0.41
DS + Surface irrigation (H_2_O_2_)	75.75 cd	34.87 cd	17.25 d	1.69 d	16.63 cd	10.95 c	210 cd	0.40
**LSD (p ≤ 0.05)**	3.68	0.65	0.27	0.13	1.90	0.72	7.53	

Values are the means of four replications and different letters indicating significant difference at p ≤ 0.05.

### 3.4 Effects on ROS production and MDA

**Figure 2:**
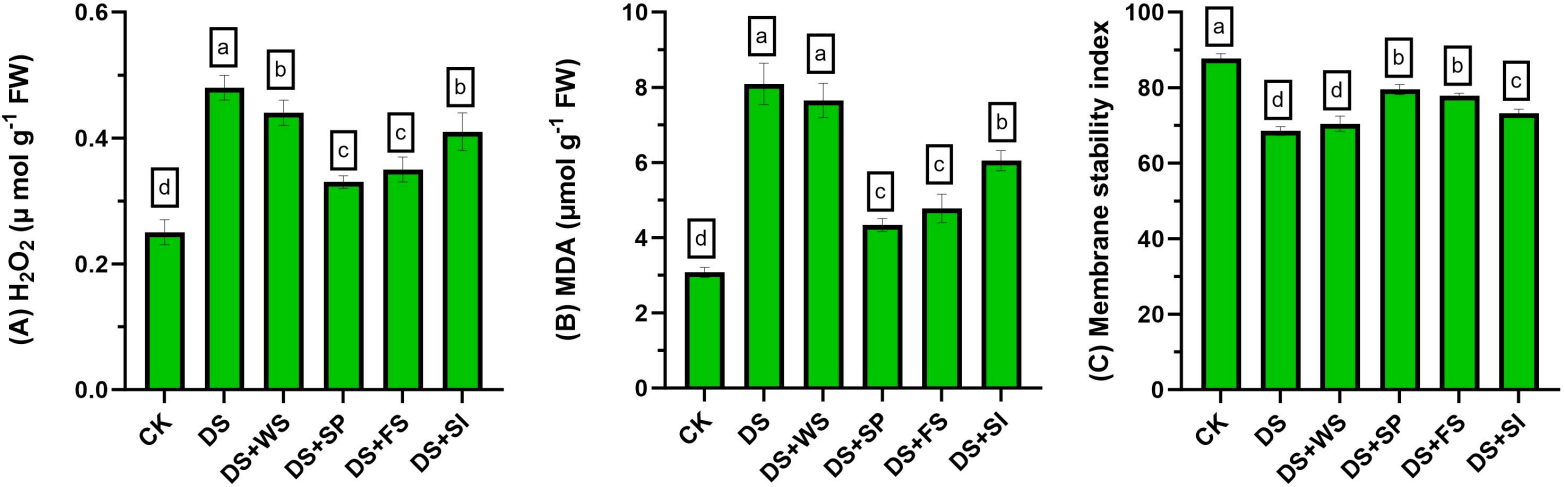
Impact of different hydrogen peroxide applications methods on **(A)** endogenous H_2_O_2_ concentration (µmol g^−1^ fresh weight), **(B)** MDA contents (µmol g^−1^ fresh weight), and **(C)** membrane stability index (MSI) of quinoa under drought stress. Values are the means of four replications and different letters indicating significant difference at p ≤ 0.05.

Drought stress significantly (p ≤ 0.05) enhanced ROS production (H_2_O_2_) and MDA contents in quinoa plants (**Figures 2A, B**) by 92% and 162%, respectively, relative to the control. Exogenously applied H_2_O_2_ significantly reduced ROS and MDA contents under drought stress. Among the application methods, seed priming followed by foliar spray showed the maximum reduction in endogenous H_2_O_2_ concentration and MDA contents. Moreover, the reduction in oxidative damages due to surface irrigation was significantly lower than other techniques of H_2_O_2_ application.

### 3.5 Effect on membrane stability index

Drought stress significantly reduced the MSI by 26% compared to control plants. Exogenous application of H_2_O_2_ through seed priming, foliar spray, and surface irrigation significantly improved MSI by 16%, 14%, and 7%, respectively, relative to control (non-treated) plants under drought stress (**Figure 2C**).

### 3.6 Effect on proline contents

**Figure 3:**
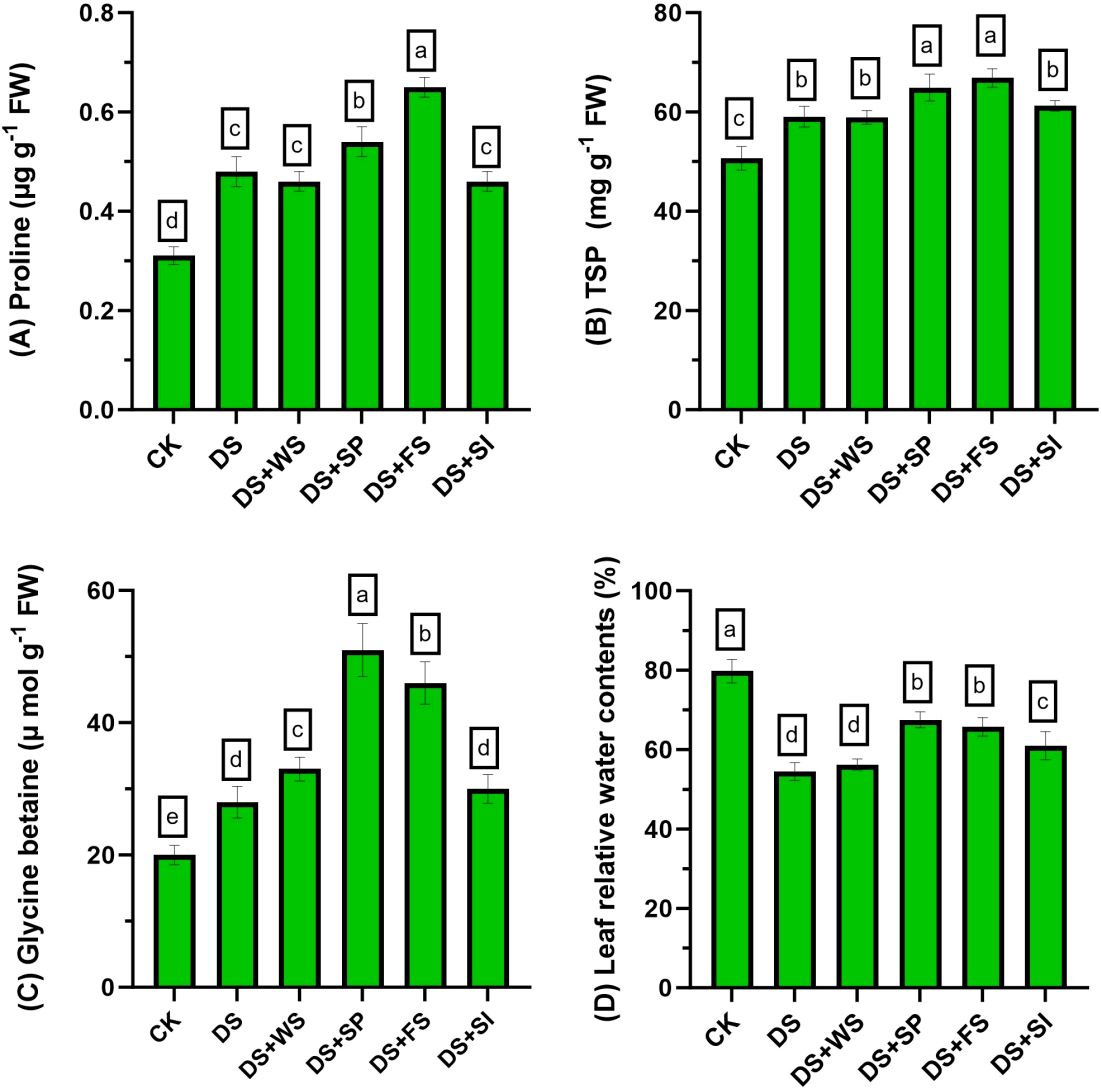
Impact of different hydrogen peroxide applications methods on **(A)** proline (µg g^−1^ fresh weight), **(B)** total soluble protein (mg g^−1^ fresh weight), **(C)** glycine betaine (µmol g^−1^ fresh weight), and **(D)** leaf relative water contents (%) of quinoa under drought stress. Values are the means of four replications and different letters indicating significant difference at p ≤ 0.05.

Drought stress markedly increased proline contents in quinoa compared to the control. Foliar and seed priming of H_2_O_2_ further improved the proline contents significantly by 35% and 12%, respectively, relative to non-treated plants under drought stress. Meanwhile, all other treatments performed similarly to the control for improving proline contents in quinoa plants under drought stress (**Figure 3A**).

### 3.7 Effect on total soluble protein contents

The TSP contents increased by 16% in quinoa under drought stress relative to control plants. Exogenously applied H_2_O_2_ further increased these contents by 10%, 13%, and 4% through seed priming, foliar spray, and surface irrigation, respectively, relative to control plants under drought stress (**Figure 3B**).

### 3.8 Effect on glycine betaine contents

Under drought stress, glycine betaine (GB) contents accumulation increased by 38% in quinoa, relative to control plants. Exogenous application of H_2_O_2_ through seed priming, foliar spray, and surface irrigation methods further improved the GB contents by 83%, 60%, and 7%, respectively, as compared to control plants under drought stress (**Figure 3C**).

### 3.9 Effect on leaf relative water contents

Drought stress considerably reduced the RWC in quinoa leaves with respect to control conditions. Exogenously applied H_2_O_2_ through seed priming, foliar spray, and surface irrigation significantly improved RWC by 24%, 22%, and 12%, respectively, relative to non-treated plants under drought stress (**Figure 3D**).

### 3.10 Effect on enzymatic antioxidants activities

**Figure 4:**
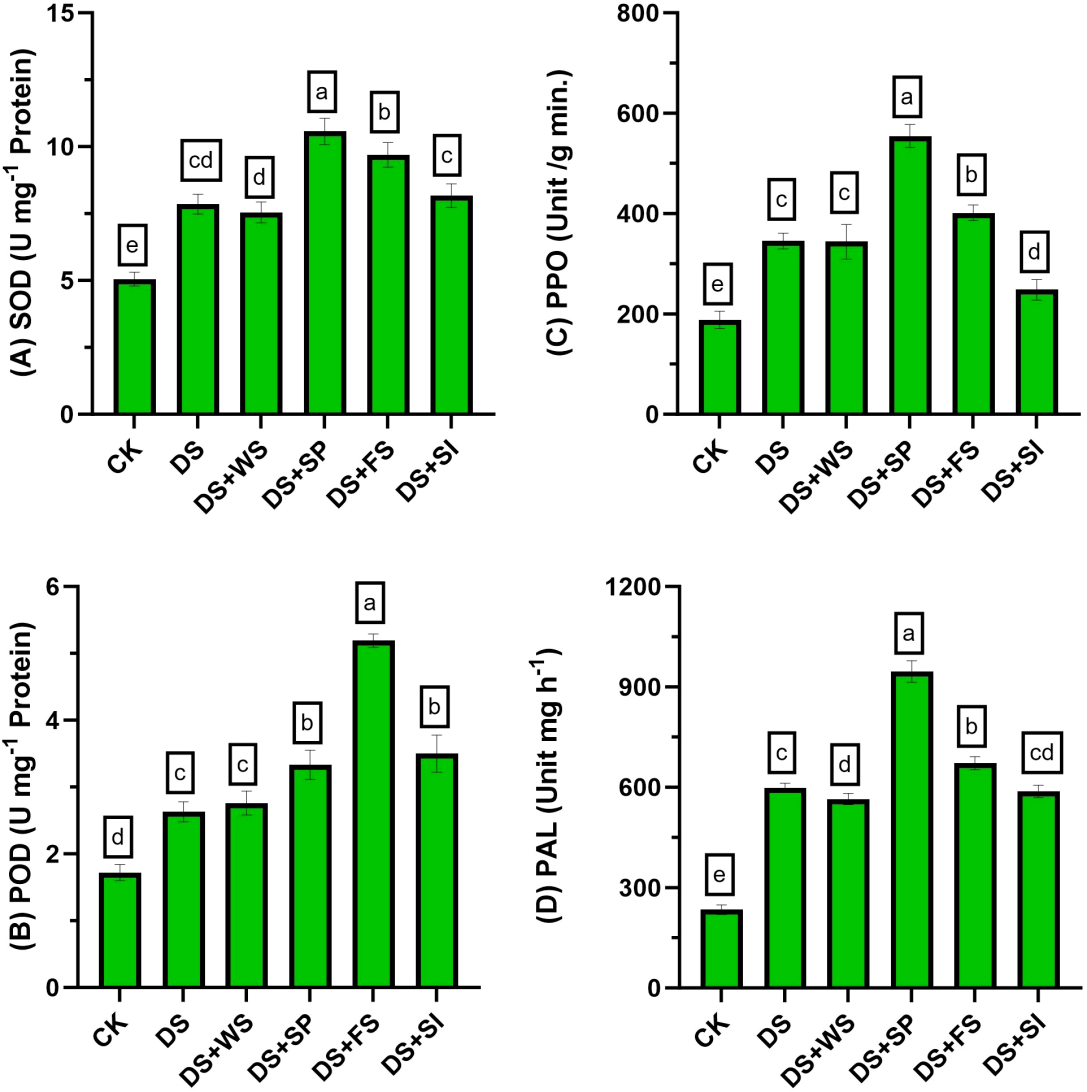
Impact of different hydrogen peroxide applications methods on **(A)** SOD (U mg^−1^ protein), **(B)** POD (U mg^−1^ protein), **(C)** PPO (unit/g min.), and **(D)** PAL (unit mg h^−1^) activity of quinoa under drought stress. Values are the means of four replications and different letters indicating significant difference at p ≤ 0.05.

Antioxidant enzyme activity markedly improved under drought in quinoa by 55%, 53%, 84%, and 156% for SOD, POD, PPO, and PAL activities, respectively, relative to control plants (**Figure 4**). Exogenously applied H_2_O_2_ further improved these enzyme activities in quinoa plants under drought stress. Among the application methods, the maximum improvement in SOD (35%), PPO (61%), and PAL (58%) activities was recorded with seed priming (H_2_O_2_), and a 97% increment in POD activity was observed with foliar-applied H_2_O_2_ relative to non-treated plants under drought stress. Surface irrigation with H_2_O_2_ was found to be statistically less effective for enhancing antioxidant activities compared to other application techniques.

### 3.11 Effect on grain yield and yield-related attributes

Quinoa grain yield and its related traits were significantly decreased (p< 0.05) under drought stress. Grain yield and 100-grain weight were reduced under drought stress by 33% and 25%, respectively, compared to the control. Exogenously applied H_2_O_2_ through either method improved these attributes under drought stress in quinoa. Nevertheless, seed priming and foliar application of H_2_O_2_ showed significantly determined grain yield (13.38 g; 12.92 g) and 100-grain weight (217 mg; 220 mg) with the highest harvest index (41), respectively, compared to surface irrigation under drought stress (**Table 4**).

### 3.12 Correlations

**Figure 5:**
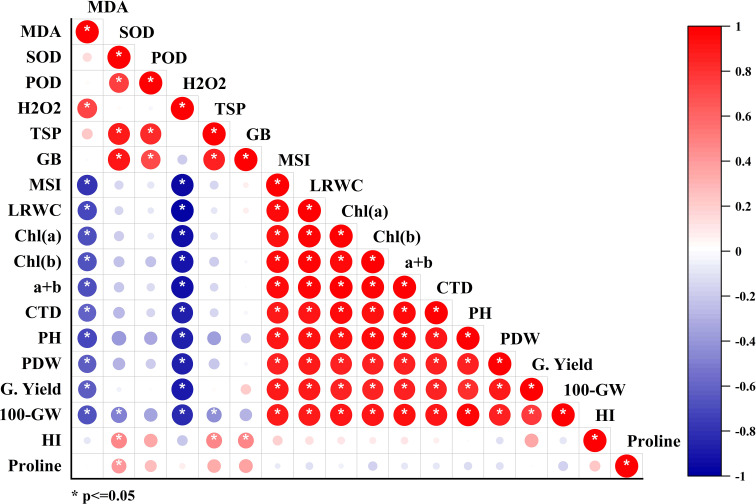
Correlations coefficients among all studied parameters.

**Figure 6:**
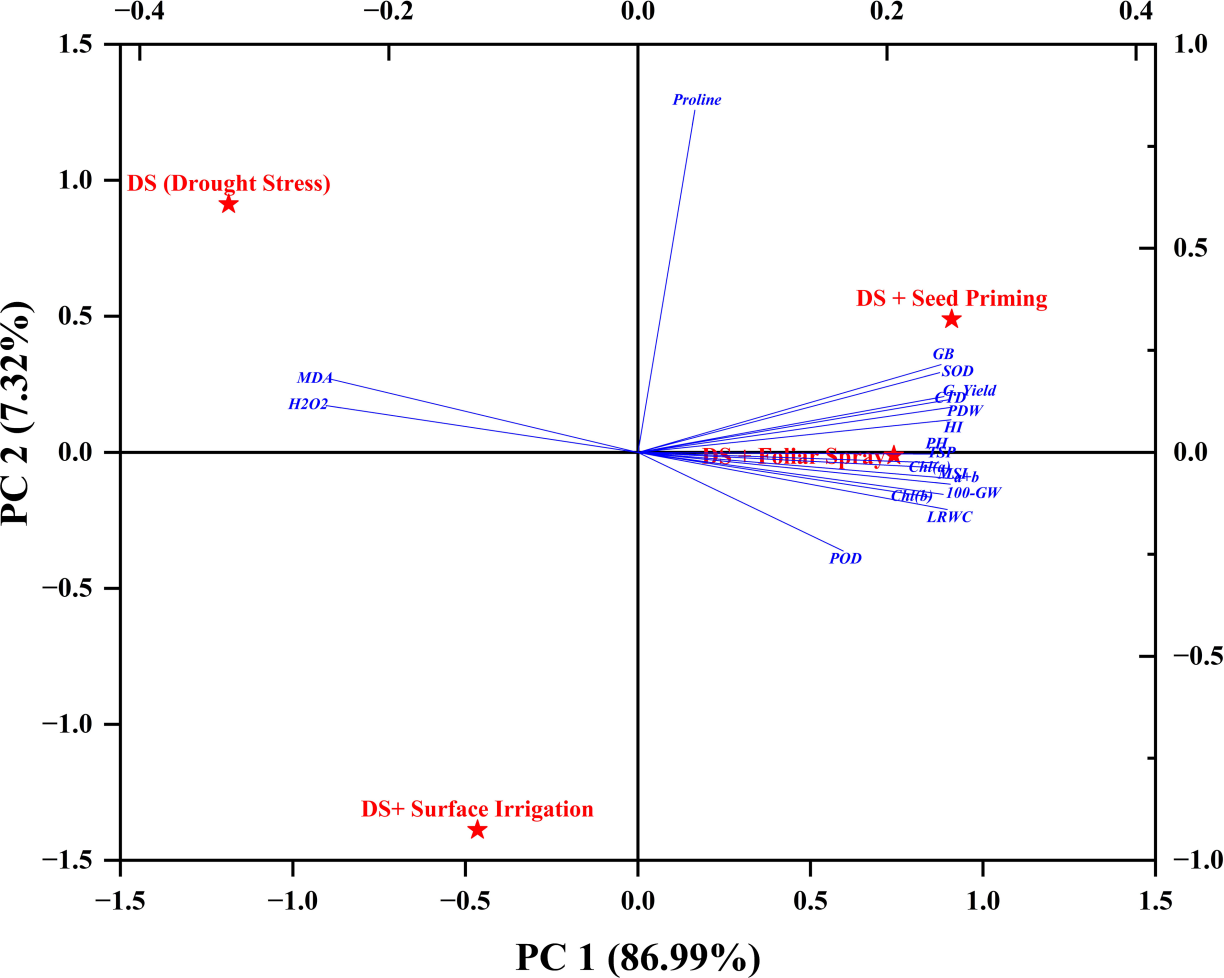
The PCA biplot showing correlations among physiochemical and yield related attributes of quinoa under different H_2_O_2_ treatments.

Correlations between all studied parameters were analyzed and presented in **Figure 5**. Total chlorophyll (*a+b*) showed a positive correlation with grain yield (R^2 =^ 0.93) and 100-grain weight (R^2 =^ 0.97) (**Figure 5**). Proline accumulation also exhibited a strong positive correlation with grain yield (R^2 =^ 0.93) and grain weight (R^2 =^ 0.93). A multivariate analysis of all studied parameters was conducted using principal component analysis (PCA). The PCA revealed that endogenous H_2_O_2_ and MDA were negatively correlated with antioxidant enzymes (SOD, POD, PPO, and PAL). However, proline, SOD, TSP, total chlorophyll, MSI, and RWC were strongly positively correlated with grain yield, and their contribution was much higher in foliar-applied H_2_O_2_ than in other application methods (**Figure 6**).

## 4 Discussion

In the present study, different modes of application were used to evaluate the impact of H_2_O_2_ on quinoa performance under drought stress. Quinoa plants are drought resilient; nonetheless, their performance reduces under drought stress (Iqbal et al., 2018a). The effect of drought on quinoa performance depends on the variety and degree of stress and is also influenced by other environmental factors. Results revealed that drought stress reduces quinoa growth and yield in the present study, and these results correspond with previous studies (Huan et al., 2022; Mahdavi Rad et al., 2022; Abbas et al., 2023; Iqbal et al., 2023). However, H_2_O_2_ application significantly improved growth attributes, chlorophyll contents, antioxidant enzyme activities, osmolyte accumulation (proline, TSP, and GB), and reduced MDA contents (**Tables 3**, **4**; **Figures 3**, **4**), ultimately resulting in increased drought tolerance. Moreover, different levels of exogenous H_2_O_2_ concentration were evaluated in a preliminary test, and results revealed that 0–15 mM concentrations were found to be safe. However, surface irrigation and foliar application showed minimum MDA contents at 5 mM and 15 mM concentrations, respectively (**Figure 1C**). The sensitivity of surface irrigation may be plausible due to rapid uptake through roots because H_2_O_2_ rapidly diffuses across the subcellular membrane and causes detrimental effects at higher concentrations (Hossain et al., 2015). These observations were further validated in detailed experiments, where surface irrigation showed higher oxidative damages under drought stress compared to redox priming and foliar application (**Figure 2B**).

**Figure 7:**
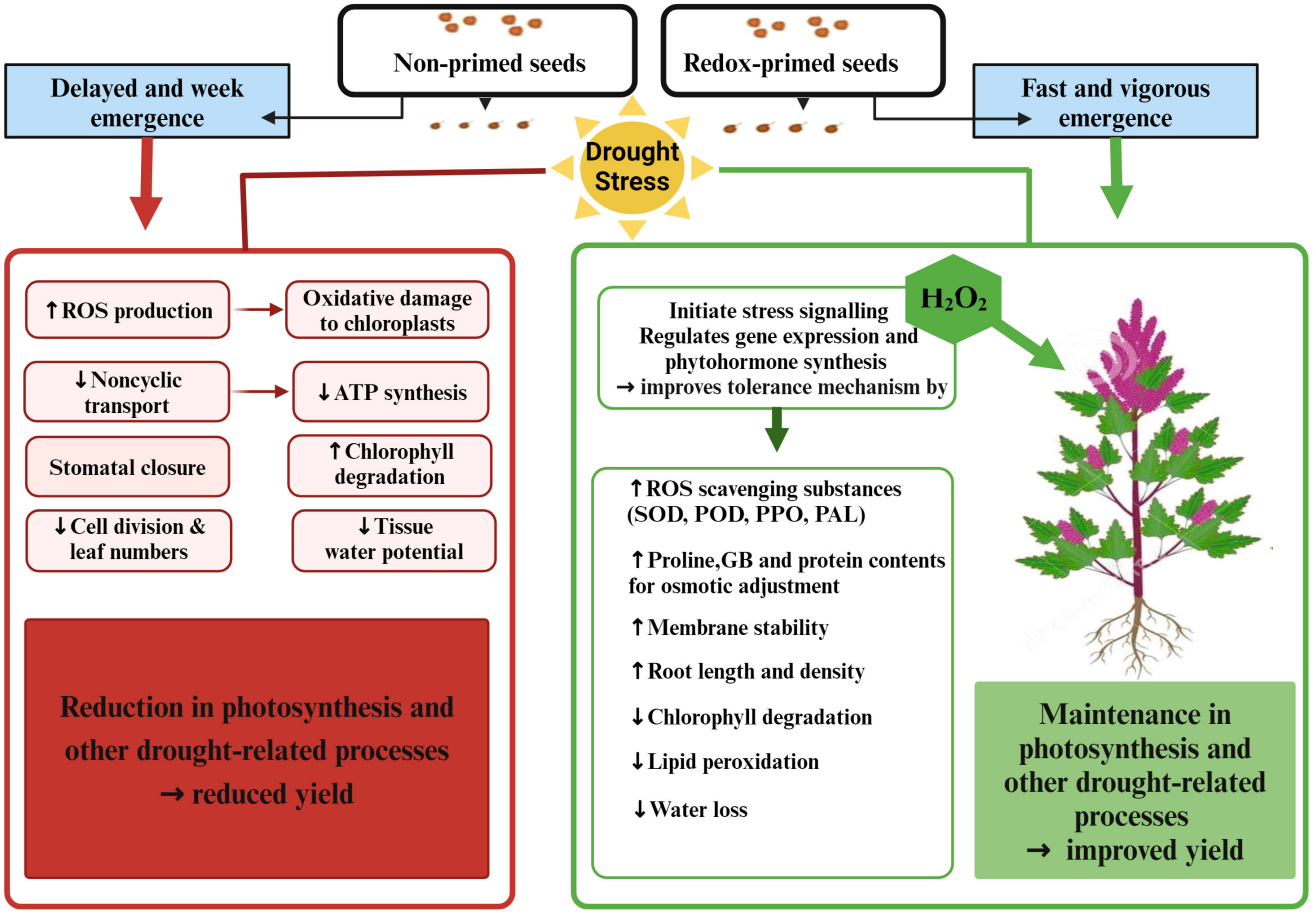
Elucidating the potential intricate mechanisms underlying drought stress and the efficacy of redox priming (H_2_O_2_) in conferring drought stress tolerance in quinoa. The illustration provides insight into the complex interplay of factors contributing to enhanced resilience through redox priming under drought conditions. Created with BioRender.com.

Among application methods, redox priming was more effective in improving quinoa performance under drought stress in terms of growth and grain yield. Seed priming with H_2_O_2_ significantly increased plant height under drought stress compared to foliar and surface irrigation methods, which might be due to early emergence with vigorous seedling growth (ur Rehman et al., 2015; Iqbal et al., 2018a). Improved emergence attributes due to H_2_O_2_ seed priming have been reported in many crop plants such as wheat, maize, sorghum, and quinoa (Ashraf et al., 2015; Iqbal et al., 2018a; Habib et al., 2021; Chattha et al., 2022; Song et al., 2023). This enhancement in emergence due to redox priming might be plausible due to oxidative modification and mobilization of stored proteins, which are considered active stimuli for emergence (Verma et al., 2015). In another study, Louis et al. (2023) reported that seed priming involves pre-exposure of seeds to mild stress, which can improve the efficiency of the DNA repair mechanism along with the activation of specific signaling proteins and transcription factors for rapid and efficient stress tolerance. Such attained stress tolerance may be retained for later developmental stages or even subsequent generations. Therefore, the improved quinoa plant growth under drought stress observed in the present study may be due to the fact that seedlings grown from redox-primed seeds acquire the ability to store memory that recalls the post-stress situation and makes the plant more tolerant to drought stress (Chen and Arora, 2013). Furthermore, redox priming led to a significant increase in root length and chlorophyll content, as indicated in **Tables 3** and **4**. This improvement may facilitate water uptake, thereby aiding in the restoration of cell turgor and ultimately enhancing photosynthesis under water-deficit conditions (Sun et al., 2016). In addition, seed priming with H_2_O_2_ significantly increased antioxidant enzymes and osmoprotectants (especially SOD, TSP, GB, PPO, and PAL) contents in quinoa leaves and reduced MDA contents under drought stress, appearing as a greater potential to improve drought resistance in quinoa compared to foliar application and surface irrigation methods (**Figure 7**).

Antioxidant metabolism, solute accumulation, and osmotic adjustment for sustained photosynthesis are key contributing factors to the tolerance mechanism. Drought stress induces structural changes in the photosynthetic machinery and causes a decreased concentration of photosynthetic pigments, as observed in the present study, which ultimately results in reduced photosynthesis. Previously, several studies have reported decreased concentrations of photosynthetic pigments due to overproduction of ROS under drought stress in different crops, including quinoa (Ashraf et al., 2015; Farooq et al., 2017; Iqbal et al., 2018a; Farooq et al., 2023).

Generally, plants respond to ROS by upregulating antioxidant enzymes and maintain osmotic balance by accumulating different metabolites (i.e., proline, TSS, and TSP) in excessive quantities under drought conditions (Jacobsen et al., 2009; Iqbal et al., 2018b; Mahdavi Rad et al., 2022). Therefore, the improvement in quinoa performance with exogenously applied H_2_O_2_ might be associated with increased stomatal conductance and improved photosynthesis (Iqbal et al., 2018a), stabilized biological membrane with improved membrane fluidity (Farooq et al., 2017), better ROS scavenging through efficient coordination with SOD and POD (Ashraf et al., 2015; Iqbal et al., 2018a), and improved osmotic adjustment through better accumulation of osmoprotectants (Terzi et al., 2014), contributing towards drought tolerance. Furthermore, exogenously applied H_2_O_2_ by either method increased proline, GB, TSP, and chlorophyll contents, which might contribute to enhanced osmotic adjustment and ultimately lead to increased grain yield under water-deficit conditions in quinoa. The increased synthesis of proline, GB, and TSP reduces drought-induced yield losses in several crops (Farooq et al., 2017), and the accumulation of these compatible solutes might have resulted in improved osmotic adjustment and a higher membrane stability index (**Figure 2C**), resulting in improved quinoa performance under drought stress.

In the present study, the increased accumulation of proline due to exogenous H_2_O_2_ was significantly positively correlated with grain weight and grain yield (**Figure 5B**). Under stress conditions, proline accumulation plays a vital role in osmotic adjustment (Hayat et al., 2012), helps in antioxidant system enhancement as evident in the present study (**Figure 4**), improves membrane integrity (**Figure 2C**), and reduces cell acidity (Hayat et al., 2012), which may lead to improved quinoa performance under drought stress. The H_2_O_2_ application significantly increased enzymatic antioxidant activity (SOD, POD, PPO, and PAL) in quinoa, which might be due to the role of H_2_O_2_ as a secondary messenger and regulates gene expressions (Hossain and Fujita, 2013; Hossain et al., 2015). Such enhanced antioxidant activities through increased oligosaccharide synthesis (Terzi et al., 2014; Ashraf et al., 2015; Guler and Pehlivan, 2016; Iqbal et al., 2018b), a decrease in ROS concentration (**Figure 1**; Hossain et al., 2015), and lipid peroxidation (**Figure 1B**; Hossain and Fujita, 2013) consequently improved the membrane stability index (**Figures 2B, C**), leading to better quinoa performance and higher grain yield under water-deficit conditions.

The endogenous H_2_O_2_ and MDA contents were also strongly negatively correlated with the antioxidant capacity of quinoa in the present study (**Figures 5**, **6**). Under drought stress, an improvement in the antioxidant defense system and a decrease in MDA content are considered important factors to maintain plant growth and yield (Sairam and Saxena, 2000). Likewise, H_2_O_2_ application increased the antioxidant enzyme activities (SOD, POD, and PAL), which were positively correlated with MSI, grain weight, and yield under drought stress in the present study (**Figures 5**, **6**). The enhanced concentration of PAL and PPO enzyme activates phenylpropanoid pathways that produce phenylpropanoid by-products like phenols and flavonoids (Farooq et al., 2017).

The derivatives of phenols and flavonoids protect cellular and subcellular membranes from oxidative damage due to their aromatic ring structure (Taiz et al., 2015), triggering ROS scavenging and revealing resistance against stress conditions. Recently, Farooq et al. (2023) also validated that higher accumulation of total phenolic contents and flavonoids provided better antioxidant capacity, resulting in reduced oxidative damage under drought and heat stress in cotton.

Exogenously applied H_2_O_2_ increased chlorophyll contents under drought stress in quinoa (**Table 4**), which was positively correlated with grain weight and yield (**Figure 5**). The improvement in chlorophyll contents due to H_2_O_2_ application under drought stress might be attributed to increased synthesis/accumulation of osmoprotectants and a stabilized biological membrane providing protection against the photosynthetic machinery and possibly increased photosynthesis (Farooq et al., 2009, 2017). It is well known that crop cultivars that potentially retain chlorophyll contents under adverse conditions have comparatively prolonged photosynthesis with higher grain-filling rates (Farooq et al., 2009; Nawaz et al., 2013), as evident from the relatively higher grain yield of quinoa under drought stress. Hence, it could be inferred that the exogenous use of H_2_O_2_, especially as seed priming, improved antioxidant metabolism, solute accumulation, and osmotic adjustment, sustaining photosynthesis in quinoa under drought stress.

## 5 Conclusion

In the present study, different modes of application were used to evaluate the impact of H_2_O_2_ on quinoa performance under drought stress. Results revealed that the reduction in plant growth and quinoa yield was significantly improved by exogenous H_2_O_2_ application under drought stress. This improvement is validated due to increased chlorophyll contents, antioxidant enzyme activities, osmolyte accumulation (proline, TSP, and GB), and reduced MDA contents (**Tables 3**, **4**; **Figures 2**, **3**), ultimately enhancing drought tolerance in quinoa. Although all studied modes of H_2_O_2_ application improved quinoa performance, surface irrigation was found to be sensitive in terms of causing oxidative damages (**Figure 1C**). Strikingly, redox priming and foliar application of H_2_O_2_ were more effective in improving quinoa performance under drought stress. Prominent plant height with maximum grain yield was found in seed priming with H_2_O_2_ compared to others under drought stress. In summary, the present study suggests further exploration to find the mechanisms behind the integration of these application methods. In essence, exogenous H_2_O_2_ application, preferably redox priming, could be chosen to decrease drought-induced performance and yield losses in quinoa. These findings are valuable for dryland agriculture, where incidents of droughts and soil salinization are common. Based on the findings, this innovative insight might provide directions in the research efforts aimed at sustainable agriculture and future food security.

## Data availability statement

The raw data supporting the conclusions of this article will be made available by the authors, without undue reservation.

## Author contributions

HI: Conceptualization, Data curation, Formal analysis, Investigation, Methodology, Project administration, Software, Validation, Visualization, Writing – original draft. CY: Funding acquisition, Project administration, Resources, Supervision, Writing – review & editing.
